# Peak Lactate Within 24 h and Mortality in Septic Shock Patients Receiving Continuous Renal Replacement Therapy: A Real-World Cohort from an Asian ICU (2018–2020)

**DOI:** 10.3390/life16010062

**Published:** 2025-12-31

**Authors:** Wei-Hung Chang, Ting-Yu Hu, Li-Kuo Kuo

**Affiliations:** 1 Department of Critical Care Medicine, MacKay Memorial Hospital, Taipei 10449, Taiwan; peacejaycool@gmail.com (W.-H.C.);; 2 Department of Medicine, Mackay Medical College, New Taipei City 25245, Taiwan

**Keywords:** peak lactate, sepsis, septic shock, continuous renal replacement therapy, blood purification, intensive care unit, mortality

## Abstract

Background: Serum lactate is a key biomarker of tissue hypoperfusion and metabolic stress in sepsis. Although lactate clearance is widely recognized, many intensive care units record only a peak lactate within 24 h (pLac-24h). The prognostic value of pLac-24h among patients receiving blood purification therapy remains unclear in Asian intensive care settings. Methods: We retrospectively analyzed the 2018–2020 ICU dataset from MacKay Memorial Hospital, Taiwan. Among 16,693 adult ICU admissions, 2506 patients received continuous renal replacement therapy (CRRT) as blood purification for severe sepsis or septic shock. Of these, 1264 (50.4%) had available pLac-24h data, and 27 (1.1%) also required extracorporeal membrane oxygenation (ECMO). The primary outcome was 28-day all-cause mortality. Multivariate logistic regression adjusted for age, sex, APACHE II score, infection source, and CRRT/ECMO use. Discrimination was evaluated by receiver operating characteristic (ROC) curves and decision-curve analysis. This analysis was conducted as a predefined sub-analysis of an institutional ICU database. Results: The mean age of the cohort was 65.7 ± 13.4 years, and 64.8% were male. Median pLac-24h was 5.1 mmol/L (IQR 3.2–8.6). The overall 28-day mortality among CRRT patients was 47.3%. Mortality rose progressively across pLac-24h quartiles (Q1–Q4: 28.9%, 39.4%, 54.7%, and 68.1%; *p* < 0.001). Each 1 mmol/L increase in pLac-24h independently predicted higher mortality (adjusted OR 1.18, 95% CI 1.10–1.26, *p* < 0.001). The area under the ROC curve for pLac-24h predicting 28-day mortality was 0.78 (95% CI 0.74–0.82), outperforming the APACHE II score (AUC 0.69, *p* = 0.02). Conclusions: In critically ill patients with septic shock undergoing CRRT, peak lactate within 24 h was a strong, independent predictor of 28-day mortality. pLac-24h offers a pragmatic, readily available prognostic indicator when serial lactate measurements are unavailable, supporting its integration into bedside risk assessment in real-world Asian ICU practice.

## 1. Introduction

Sepsis and septic shock remain major causes of mortality in intensive care units (ICUs) worldwide despite advances in early recognition, antimicrobial therapy, and organ support [1,2,3]. The Sepsis-3 consensus redefined septic shock as a dysregulated host response with profound circulatory and metabolic abnormalities that substantially increase the risk of death [4]. Current guidelines emphasize not only prompt initiation of resuscitation but also dynamic assessment of tissue perfusion and metabolic recovery [5,6].

Among available biomarkers, serum lactate remains central to the management of septic shock. Elevated lactate reflects impaired oxygen utilization, microcirculatory failure, and metabolic stress [7,8]. However, absolute lactate levels are influenced by hepatic clearance, adrenergic stimulation, and extracorporeal therapies [9,10]. For this reason, lactate clearance—the relative reduction over time—has traditionally been used as a dynamic prognostic marker [11]. Recent reviews and international guidelines continue to emphasize the prognostic relevance of lactate dynamics in septic shock, including in patients receiving advanced organ support [5,6,12,13,14]. Nevertheless, many ICUs, especially in real-world practice, lack serial lactate timepoints and instead archive only a single peak lactate value within the first 24 h (pLac-24h) after ICU admission [15,16]. The clinical implications of this common data pattern have not been systematically evaluated, particularly in patients receiving blood purification.

Continuous renal replacement therapy (CRRT) is frequently used in septic shock for renal failure and metabolic control [17,18]. While CRRT may partially influence lactate kinetics through acid–base regulation or metabolic clearance, elevated lactate levels in this population still indicate persistent tissue hypoperfusion and systemic inflammation [19]. High lactate concentrations have been associated with increased mortality among critically ill patients, yet the prognostic value of pLac-24h specifically in CRRT-treated septic shock patients remains uncertain [20,21,22].

In Asian ICUs—where Gram-negative sepsis predominates and access to advanced organ support is broad under universal health coverage—real-world data are essential to optimize risk stratification and guide timely intervention [23,24]. Taiwan’s national healthcare system provides wide access to CRRT, offering an opportunity to evaluate the predictive role of pLac-24h in a large, homogeneous cohort of patients with severe sepsis or septic shock.

This study aimed to assess the association between peak lactate within 24 h of ICU admission and 28-day mortality among adult patients with septic shock undergoing CRRT. We further evaluated whether pLac-24h adds incremental prognostic value beyond established clinical severity indices, such as the APACHE II score, to support early risk stratification and management decisions in real-world Asian ICU practice.

Among patients with septic shock admitted to the ICU, acute kidney injury requiring continuous renal replacement therapy (CRRT) occurs in approximately 20–40% of cases and is associated with particularly high short-term mortality. Despite advances in organ support, reliable early prognostic markers in this high-risk subgroup remain limited, especially in real-world settings where serial lactate measurements are not consistently available.

Therefore, clarifying the prognostic value of peak lactate within the first 24 h (pLac-24h) in septic shock patients receiving CRRT addresses an important gap in current clinical knowledge and may inform early risk stratification in routine ICU practice.

## 2. Materials and Methods

### 2.1. Study Design and Setting

This retrospective observational cohort study was conducted as a predefined sub-analysis of an IRB-approved adult intensive care unit (ICU) database project at MacKay Memorial Hospital. The umbrella project, entitled “Construction of intelligent system for improving medical care in adult intensive care units: artificial intelligence models for early prediction of sepsis using unstructured medical data” (IRB No. 21MMHIS430e; approval date: 14 January 2022), maintains a de-identified database of all adult ICU admissions for quality improvement and research purposes. The hypothesis regarding the prognostic role of peak lactate within 24 h (pLac-24h) was defined prior to analysis. The primary research question was whether peak lactate within 24 h of ICU admission independently predicts 28-day mortality in septic shock patients receiving CRRT. We hypothesized that higher pLac-24h values would be associated with increased short-term mortality, even after adjustment for established severity indices.

### 2.2. Study Population

Among 16,693 unique ICU admissions during the study period, 2506 adult patients (≥20 years) who received continuous renal replacement therapy (CRRT) for severe sepsis or septic shock were screened. CRRT was defined as blood purification therapy documented by the “renal replacement therapy” variable in the dataset (indicator = 1). CRRT was initiated for sepsis-associated acute kidney injury or for metabolic control according to predefined dataset variable definitions. Patients were excluded if they had missing mortality data, were readmitted during the same hospitalization, or lacked lactate measurements within 24 h of ICU admission. After exclusions, 1264 patients (50.4%) with available 24 h peak lactate data were included in the final analysis. Lactate measurements within the first 24 h were unavailable in some patients due to delayed ICU admission, early mortality before laboratory sampling, transfer from other hospitals without complete laboratory records, or clinical prioritization of urgent organ support before routine metabolic assessment. Lactate measurement was part of routine sepsis evaluation rather than being restricted to the most critically ill patients; therefore, missing data were primarily attributable to logistical and timing-related factors rather than selective testing based on illness severity.

### 2.3. Data Collection

All variables used in this study were extracted from the de-identified adult ICU database established under the IRB-approved project “Construction of intelligent system for improving medical care in adult intensive care units: artificial intelligence models for early prediction of sepsis using unstructured medical data” (IRB No. 21MMHIS430e). The database includes all adult ICU admissions from the participating sites (Taipei and Tamsui MacKay Memorial Hospitals) and contains routinely collected clinical information for research and quality improvement purposes. The requirement for informed consent was waived because only de-identified retrospective data were analyzed.

Demographic and clinical variables were extracted from the electronic database, including:

Baseline characteristics: age, sex, body weight, comorbidities, infection source, and microbiological profile.

Severity indicators included the Acute Physiology and Chronic Health Evaluation II (APACHE II) score and the Sequential Organ Failure Assessment (SOFA) score, both calculated at ICU admission according to standard definitions.

### 2.4. Exposure Variable

The primary exposure was peak lactate concentration within 24 h of ICU admission (pLac-24h), recorded as the highest lactate value (mmol/L) measured during the first 24 h. The timing of lactate sampling relative to CRRT or ECMO initiation could not be fully ascertained in all cases. pLac-24h was analyzed as a continuous variable (per 1 mmol/L increment) and by quartiles (Q1–Q4) to assess dose–response relationships. Analyses of lactate cutoffs were performed as exploratory sensitivity analyses.

### 2.5. Outcome Measures

The primary outcome was 28-day all-cause mortality after ICU admission. Secondary outcomes included ICU and hospital mortality, ICU/hospital length of stay (LOS), and the duration of organ-support therapies (CRRT and ECMO). Ninety-day survival was analyzed as a secondary time-to-event outcome.

### 2.6. Statistical Analysis

Continuous variables are presented as mean ± standard deviation (SD) or median (interquartile range, IQR) according to distribution; categorical variables are expressed as counts (%) and compared using the χ^2^ test or Fisher’s exact test as appropriate. Between-group comparisons across pLac-24h quartiles used one-way ANOVA or Kruskal–Wallis tests.

Associations between pLac-24h and 28-day mortality were evaluated using multivariate logistic regression. Covariates were selected a priori based on clinical relevance and established associations with sepsis mortality. Multicollinearity was assessed using variance inflation factors (VIF), with all variables demonstrating acceptable collinearity (VIF < 5). The model adjusted for age, sex, APACHE II score, infection source, CRRT duration, and ECMO use. Model discrimination was assessed using receiver operating characteristic (ROC) curves and area under the curve (AUC) analysis. Improvement in predictive performance was tested by comparing models with and without pLac-24h (using ΔAUC and DeLong’s test). Clinical net benefit was estimated via decision-curve analysis (DCA).

All statistical tests were two-tailed with a significance threshold of *p* < 0.05. Analyses were performed using IBM SPSS Statistics v26.0 (IBM Corp., Armonk, NY, USA) and R software v4.2 (R Foundation for Statistical Computing, Vienna, Austria). Due to the retrospective nature of the database, detailed vasopressor dosages (e.g., norepinephrine-equivalent dose), cumulative fluid balance, baseline renal function (e.g., baseline creatinine or CKD stage), timing of CRRT initiation relative to ICU admission, and certain major comorbidities (e.g., cirrhosis) were not consistently available and therefore could not be included in the primary adjusted model.

## 3. Results

### 3.1. Study Population and Baseline Characteristics

**Figure 1:**
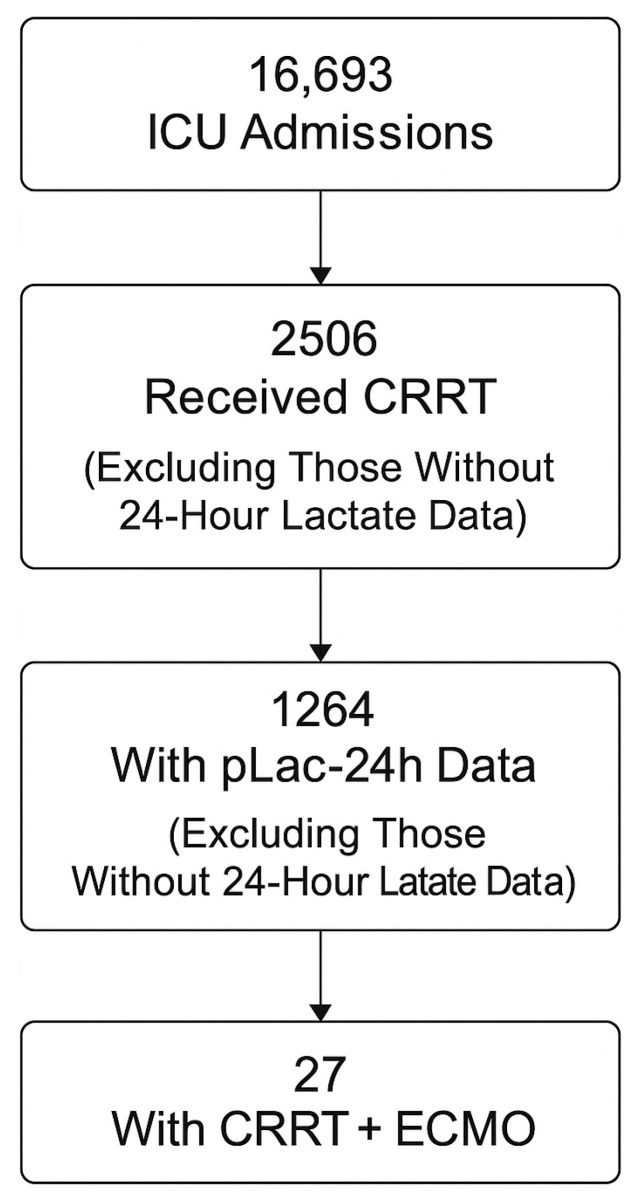
Study flow diagram. Exclusion criteria included missing mortality data, readmission during the same hospitalization, and absence of lactate measurements within 24 h of ICU admission.

During the 2018–2020 study period, 16,693 ICU admissions were recorded. Among them, 2506 adults received continuous renal replacement therapy (CRRT) for severe sepsis or septic shock. After excluding cases without recorded lactate values, 1264 patients (50.4%) with available peak lactate within 24 h (pLac-24h) were analyzed (Figure 1). Baseline demographic and clinical characteristics were broadly comparable between patients with available pLac-24h data and those without, with no clinically meaningful differences in age, sex, or severity scores (Table 1). The mean age was 65.7 ± 13.4 years, and 64.8% were male. The median APACHE II score at ICU admission was 26 (IQR 21–32). The most common infection sources were pneumonia (31.2%), intra-abdominal infection (18.5%), and urinary tract infection (16.9%). Gram-negative pathogens predominated, with Escherichia coli and Klebsiella pneumoniae being most frequent. Among all CRRT recipients, 27 patients (1.1%) also required extracorporeal membrane oxygenation (ECMO). Baseline characteristics stratified by 28-day survival are summarized in Table 1.

### 3.2. Distribution of Peak Lactate (pLac-24h)

The overall median pLac-24h was 5.1 mmol/L (IQR 3.2–8.6). Patients were divided into quartiles:

Q1: ≤3.0 mmol/L,

Q2: 3.1–5.0 mmol/L,

Q3: 5.1–8.0 mmol/L, and

Q4: >8.0 mmol/L.

**Figure 2:**
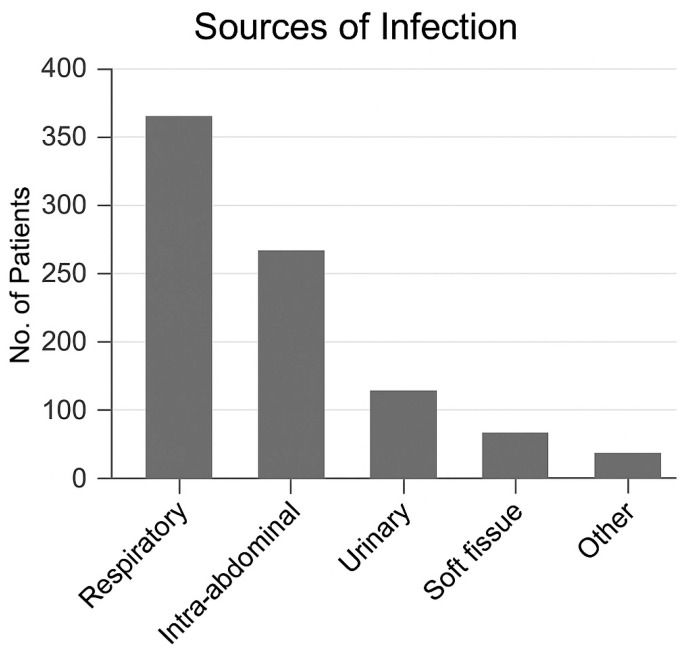
Distribution of peak lactate by survival status.

Higher pLac-24h values were associated with older age, higher APACHE II scores, and increased requirements for vasoactive agents and organ supports (*p* < 0.01 for all). The distribution of pLac-24h by survival status is illustrated in Figure 2.

### 3.3. Association Between pLac-24h and 28-Day Mortality

The overall 28-day mortality among CRRT patients was 47.3% (598/1264). Mortality increased stepwise across pLac-24h quartiles: Q1 28.9%, Q2 39.4%, Q3 54.7%, Q4 68.1% (*p* < 0.001). In multivariate logistic regression adjusted for age, sex, APACHE II score, infection source, CRRT duration, and ECMO use, each 1 mmol/L increase in pLac-24h was independently associated with an 18% rise in the odds of death (adjusted OR 1.18, 95% CI 1.10–1.26, *p* < 0.001). When CRRT duration was excluded from the multivariable model, the association between pLac-24h and 28-day mortality remained materially unchanged. Compared with Q1, patients in Q4 had an adjusted OR of 3.42 (95% CI 2.21–5.28, *p* < 0.001). These findings are detailed in Table 2.

In sensitivity analyses stratified by mechanical ventilation status, the association between pLac-24h and 28-day mortality remained directionally consistent in both ventilated and non-ventilated patients. No clinically meaningful effect modification was observed.

Subgroup analyses across ECMO use, major infection sources, and APACHE II strata demonstrated directionally consistent associations between pLac-24h and 28-day mortality, without evidence of clinically meaningful effect modification.

### 3.4. Model Performance and Predictive Value

**Figure 3:**
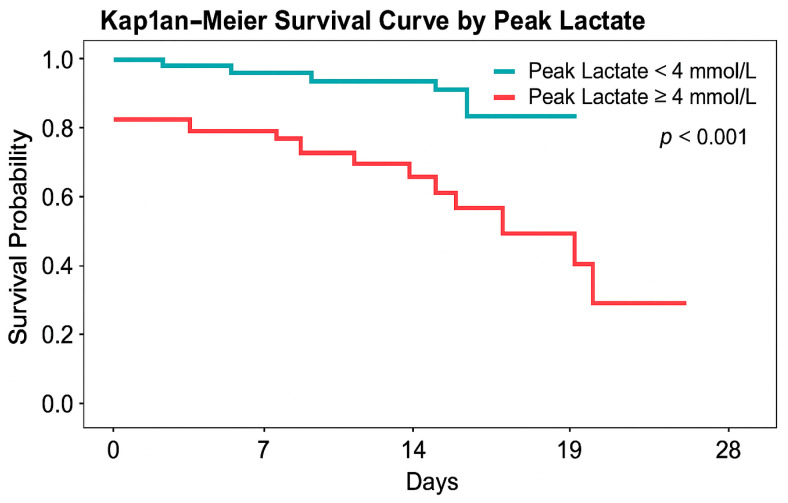
Kaplan–Meier survival curves stratified by peak lactate within 24 h (pLac-24h). Patients were grouped according to clinically relevant lactate thresholds. Clinically relevant lactate thresholds (4 and 8 mmol/L) were evaluated in prespecified analyses. Survival was significantly lower in patients with pLac-24h ≥ 4 mmol/L (log-rank *p* < 0.001).

The receiver operating characteristic (ROC) curve for pLac-24h predicting 28-day mortality yielded an area under the curve (AUC) = 0.78 (95% CI 0.74–0.82), indicating good discrimination (Figure 3).

**Figure 4:**
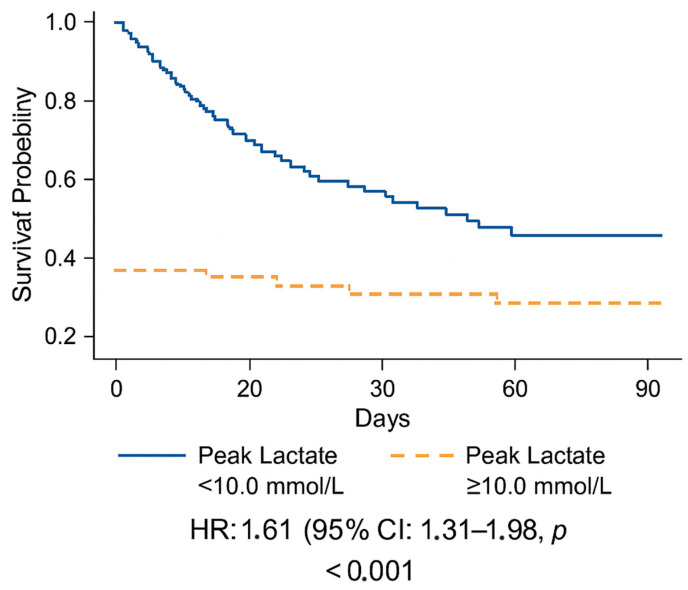
Decision-curve analysis (DCA) of clinical net benefit.

When combined with the APACHE II score, overall model performance improved (AUC 0.83 vs. 0.69 for APACHE II alone; ΔAUC = 0.14, *p* = 0.02). Decision-curve analysis demonstrated a consistent net clinical benefit for the combined model across a wide range of decision thresholds (Figure 4).

### 3.5. Secondary Outcomes

Patients in the highest pLac-24h quartile (Q4) had longer ICU stays (median 12.4 vs. 7.6 days, *p* = 0.01 *) and higher ICU and hospital mortality (ICU 63.7% vs. 31.5%; hospital 70.2% vs. 36.8%; *p* < 0.001). CRRT duration was significantly longer among non-survivors (median 8 vs. 5 days, *p* = 0.03 *). ECMO use was more frequent in non-survivors (2.8% vs. 1.0%), though not statistically significant (*p* = 0.22 *). A summary of outcomes by pLac-24h quartiles is shown in Table 3.

### 3.6. Key Findings

pLac-24h independently predicted 28-day mortality in CRRT-treated septic shock.

Higher pLac-24h values were associated with prolonged ICU stay and greater resource utilization.

pLac-24h significantly improved discrimination beyond APACHE II alone.

These results confirm that a single lactate measurement within 24 h retains strong prognostic power in real-world Asian ICU practice, even without serial lactate data.

## 4. Discussion

This study aimed to evaluate whether peak lactate within 24 h of ICU admission serves as an independent prognostic marker for short-term mortality in septic shock patients receiving CRRT. Mortality increased in a stepwise manner across pLac-24h quartiles, and each 1 mmol/L rise in pLac-24h was associated with an 18% increase in adjusted odds of death. Incorporating pLac-24h into severity models improved prognostic discrimination and clinical net benefit compared with the APACHE II score alone.

### 4.1. Comparison with Previous Studies

Our findings are consistent with prior investigations showing that lactate level is a robust marker of sepsis severity and outcome [25,26,27]. Previous studies emphasized lactate clearance as a dynamic variable [28,29,30], yet sequential sampling is not always feasible in routine practice, especially when extracorporeal therapy influences fluid balance and sampling timing. The current analysis extends prior evidence by confirming that a single early lactate measurement retains strong prognostic value in a large Asian ICU cohort, even in patients receiving CRRT.

CRRT may modestly influence lactate kinetics through acid–base regulation and solute exchange, but it does not substantially eliminate lactate because of molecular size and production–clearance equilibrium [31,32,33]. Persistent hyperlactatemia during CRRT therefore likely reflects ongoing tissue hypoxia, microcirculatory dysfunction, or impaired mitochondrial metabolism rather than an artifact of extracorporeal removal [34]. Similar graded relationships between lactate concentration and mortality have been reported in international sepsis registries, confirming its biological and prognostic relevance [12,13].

### 4.2. Clinical Implications

The present findings highlight peak lactate within 24 h (pLac-24h) as a simple and pragmatic prognostic indicator in real-world ICU practice. Unlike dynamic lactate clearance metrics, pLac-24h relies on a single routinely available measurement and therefore aligns closely with everyday clinical workflows, particularly in critically ill patients requiring complex organ support.

The clinical value of pLac-24h becomes especially evident in settings where serial lactate measurements are unavailable or inconsistently obtained. In many ICUs, including high-acuity referral centers, logistical constraints, extracorporeal therapies, and urgent resuscitative priorities limit the feasibility of repeated lactate sampling. Under such circumstances, pLac-24h provides a robust surrogate of early metabolic stress and persistent tissue hypoperfusion, allowing clinicians to retain meaningful prognostic insight despite incomplete temporal data.

Consistent with these findings, decision-curve analysis suggested a favorable clinical net benefit for models incorporating pLac-24h across a range of reasonable decision thresholds. Decision-curve analysis was used to estimate potential clinical net benefit across a range of clinically relevant threshold probabilities rather than to define a single actionable cutoff.

### 4.3. Strengths and Limitations

The major strengths of this study include a large real-world cohort drawn from a tertiary referral ICU under Taiwan’s universal health-care system, minimizing selection bias and reflecting typical Asian ICU practice. Comprehensive electronic capture of CRRT parameters and mortality endpoints permitted robust modeling of metabolic predictors.

However, several limitations should be acknowledged.

First, missing lactate data in approximately half of CRRT-treated patients reflects real-world ICU practice and may introduce potential selection bias; nevertheless, the analyzed cohort remained sufficiently large to ensure statistical power, and baseline comparisons showed no major demographic differences from excluded cases.

Second, only the highest lactate value within the first 24 h was available, preventing direct assessment of true lactate clearance and dynamic metabolic trends. Formal assessment of non-linearity or model calibration was beyond the scope of this retrospective analysis. Although pLac-24h reflects early metabolic stress, we could not completely exclude the possibility that some lactate measurements occurred after initiation of CRRT or ECMO, which may have influenced measured lactate levels.

Third, the retrospective single-center design limits causal inference and generalizability beyond similar healthcare systems.

Fourth, certain potentially relevant confounders—such as vasoactive dosage, CRRT initiation timing, and fluid balance—were unavailable, introducing possible residual bias. In addition, CRRT duration may be influenced by survival time and overall illness severity, and its inclusion as a linear predictor in regression models may introduce survivor or collider bias.

### 4.4. Future Directions

Future research should validate pLac-24h thresholds across multiple Asian centers and explore their integration with other dynamic metrics—such as lactate clearance, VIS trajectories, and inflammatory biomarkers—to refine sepsis prognostication [35,36,37,38,39]. Combining metabolic and hemodynamic information may yield composite scoring systems that guide individualized timing of CRRT initiation or discontinuation. Prospective multicenter studies with standardized lactate sampling and detailed treatment records are needed to confirm these findings and evaluate cost-effectiveness. These findings should be interpreted as hypothesis-generating and warrant external validation [40].

## 5. Conclusions

In this large, real-world ICU cohort from Taiwan, peak lactate within 24 h of admission (pLac-24h) was an independent predictor of 28-day mortality among septic shock patients receiving continuous renal replacement therapy. Each 1 mmol/L increase in pLac-24h was associated with an approximately 18% higher risk of death. Incorporating pLac-24h into standard severity models significantly improved prognostic discrimination beyond APACHE II, highlighting its practicality as a single-time-point metabolic marker when serial lactate monitoring is unavailable. Routine evaluation of pLac-24h may enhance early risk stratification and guide timely intervention in real-world Asian ICU practice.

## Figures and Tables

**Table 1:** Baseline characteristics stratified by 28-day survival.

Variable	Survivors (n = 666)	Non-Survivors (n = 598)	*p*-Value
Age, years (mean ± SD)	63.8 ± 13.8	67.9 ± 12.5	0.001
Male, n (%)	421 (63.2)	400 (66.9)	0.18
APACHE II score, median (IQR)	24 (20–30)	28 (23–34)	<0.001
Infection source, n (%)			
Pneumonia	202 (30.3)	192 (32.1)	0.52
Intra-abdominal infection	124 (18.6)	110 (18.4)	0.94
Urinary tract infection	121 (18.2)	92 (15.4)	0.19
Other/mixed	219 (32.9)	204 (34.1)	0.67
CRRT duration, days, median (IQR)	5 (3–8)	8 (5–11)	0.03
ECMO use, n (%)	5 (0.8)	22 (3.7)	0.004
Mechanical ventilation, n (%)	653 (98.0)	595 (99.5)	0.04
ICU length of stay, days, median (IQR)	7.6 (4.0–14.2)	12.4 (6.1–20.3)	<0.001

Note: Continuous variables are expressed as mean ± standard deviation (SD) or median (interquartile range, IQR) as appropriate; categorical variables are shown as number (%). Statistical comparisons were performed using Student’s *t*, Mann–Whitney U, or χ^2^ tests.

**Table 2:** Multivariate logistic regression for predictors of 28-day mortality.

Variable	Adjusted OR	95% CI	*p*-Value
Age (per year)	1.02	1.01–1.03	0.001
Male sex	1.12	0.88–1.43	0.36
APACHE II (per point)	1.05	1.03–1.07	<0.001
pLac-24h (per mmol/L)	1.18	1.10–1.26	<0.001
CRRT duration (per day)	1.04	1.01–1.08	0.03
ECMO use (yes vs. no)	1.92	0.92–4.01	0.08
Pneumonia source (vs. other)	1.21	0.94–1.57	0.14

**Table 3:** Outcomes According to pLac-24h quartiles.

Variable	Q1 (≤3.0 mmol/L)	Q2 (3.1–5.0 mmol/L)	Q3 (5.1–8.0 mmol/L)	Q4 (>8.0 mmol/L)	*p*-Trend
No. of patients	316	316	316	316	—
28-day mortality, %	28.9	39.4	54.7	68.1	<0.001
ICU mortality, %	31.5	42.1	57.3	63.7	<0.001
Hospital mortality, %	36.8	46.5	60.5	70.2	<0.001
ICU LOS, days, median (IQR)	7.2 (4.1–13.0)	8.1 (4.5–14.8)	10.7 (6.3–17.4)	12.4 (7.1–19.9)	0.01
Hospital LOS, days, median (IQR)	20.3 (11.0–36.5)	22.8 (12.5–39.7)	25.4 (14.3–42.6)	26.7 (14.7–46.2)	0.04
CRRT duration > 7 days, %	32.4	38.7	46.8	58.3	0.03
ECMO use, %	0.6	0.9	1.5	3.8	0.12

## Data Availability

The data that support the findings of this study are not publicly available due to institutional and legal restrictions related to patient privacy. De-identified data may be made available from the corresponding author upon reasonable request and with prior approval from the Institutional Review Board of MacKay Memorial Hospital.
